# Multiple routes for compound word processing in the brain: Evidence from EEG^☆^

**DOI:** 10.1016/j.bandl.2013.04.002

**Published:** 2013-08

**Authors:** Lucy J. MacGregor, Yury Shtyrov

**Affiliations:** Medical Research Council (MRC) Cognition and Brain Sciences Unit, 15 Chaucer Rd., Cambridge CB2 7EF, UK

**Keywords:** Compounds, Dual-route, Speech, Language, ERPs, MMN

## Abstract

Are compound words represented as unitary lexical units, or as individual constituents that are processed combinatorially? We investigated the neuro-cognitive processing of compounds using EEG and a passive-listening oddball design in which lexical access and combinatorial processing elicit dissociating Mismatch Negativity (MMN) brain-response patterns. MMN amplitude varied with compound frequency and semantic transparency (the clarity of the relationship between compound and constituent meanings). Opaque compounds elicited an enhanced ‘lexical’ MMN, reflecting stronger lexical representations, to high- vs. low-frequency compounds. Transparent compounds showed no frequency effect, nor differed to pseudo-compounds, reflecting the combination of a reduced ‘syntactic’ MMN indexing combinatorial links, and an enhanced ‘lexical’ MMN for real-word compounds compared to pseudo-compounds. We argue that transparent compounds are processed combinatorially alongside parallel lexical access of the whole-form representation, but whole-form access is the dominant mechanism for opaque compounds, particularly those of high-frequency. Results support a flexible dual-route account of compound processing.

## Highlights

• MMN response dissociates lexical access and combinatorial processing of compounds.
• Compound lexical frequency and meaning transparency affect the MMN.
• Larger MMN for high vs. low-frequency opaque compounds.
• No MMN frequency effects for transparent compounds or differences to pseudo-compounds.
• Results support a parallel dual-route account of compound word processing.

## 1 Introduction

The representation and processing of compound words, and morphologically complex words more generally, remains a controversial topic in psycholinguistics. Are compound words represented and processed as unitary lexical units as proposed by full-listing models (Bybee, 1995), or only as individual constituents that are analysed via a combinatorial mechanism as proposed by full-parsing models (Libben, Derwing, & de Almeida, 1999; Taft, 2004; Taft & Forster, 1976)? Alternatively, both mechanisms may be invoked as suggested by dual-route models (Baayen, Dijkstra, & Schreuder, 1997; Isel, Gunter, & Friederici, 2003; Koester, Gunter, & Wagner, 2007; Koester, Gunter, Wagner, & Friederici, 2004; Koester, Holle, & Gunter, 2009; Sandra, 1990; Zwitserlood, 1994). One feature that may affect representation and processing is semantic transparency, the clarity of the relationship between the meaning of the compound and that of its constituents. The meaning of fully transparent compound words (e.g. *homework*, *workman*) can be understood from the combination of the meanings of their individual constituents (*home* + *work*, *work* + *man*). Therefore, in principle, transparent compounds do not require a distinct lexical representation but may be processed via a mechanism akin to syntactic rules linking words in sentences. By contrast, the meaning of opaque compounds cannot be derived from their constituents (e.g. *framework*, *strawman*) and thus may require dedicated whole-form lexical storage. A second potentially important factor is that of the overall lexical frequency: more frequent words are more likely to benefit from readily available whole-form storage (which, in turn, may be more likely to develop as a result of frequent use), whereas less frequently used compounds might have to be processed through a combinatorial mechanism. Here we investigate the representation and processing of spoken compound words using a passive-listening oddball paradigm. By capitalising on the existence of different patterns of Mismatch Negativity (MMN) amplitudes depending on whether the link between first and second constituents is lexical or syntactic, we ask whether hearing the second constituent of a semantic or transparent compound triggers access to a whole compound representation or combinatorial processing. Before describing our experimental approach in more detail, we review the existing data on compound processing.

To explore whether the meanings of individual constituents are accessed during compound word processing, a number of behavioural studies used a semantic priming paradigm. It was shown that, for transparent but not opaque compounds, lexical decision times to two-constituent^1^ compound words were speeded up by a preceding prime that was semantically related to either the first or second constituent of the target compound word (Sandra, 1990; Zwitserlood, 1994). From this it was argued that individual constituent semantics were accessed only for transparent compounds, suggesting the possibility of combinatorial processing only for transparent compounds, but a direct whole-form access route for opaque compounds. Further evidence for access to constituent meanings of transparent but not opaque compounds comes from a cross-modal priming study in which visually presented transparent compound words were primed by the prior auditory presentation of both first and second compound constituents and vice versa, but no such effects were observed for opaque compounds (Zhou & Marslen-Wilson, 2000). In line with these findings, another cross-modal semantic priming study showed that the first constituents of German spoken compounds primed visually presented targets only when the second constituent was transparent, but not when it was opaque, suggesting that activation of both constituents is dependent on the transparency of the second constituent (Isel et al., 2003).

Access to compound constituents has also been studied neurophysiologically, using event-related potentials (ERPs). Here, the evidence for activation of constituent semantics is mixed. In one study using a cross-modal semantic priming paradigm the amplitude of the N400 electrophysiological brain response to spoken English compounds was modulated by the relatedness of preceding pictures to either of the compound constituents. This finding indicates activation of both constituents (Pratarelli, 1995), although reaction times did not show semantic priming effects. Notably, however, this study did not control for transparency, which does not allow us to conclude whether or not the observed effects occur for different subtypes of compounds.

Despite the widespread use of the semantic priming paradigm, it has been argued (Koester et al., 2007) that, on their own, semantic priming effects between constituents and compounds are not conclusive evidence for combinatorial processing, because they could be driven by pure semantic relatedness; the lack of semantic priming for opaque compounds could simply reflect the unrelatedness between the meaning of the compound and its constituents rather than the absence of an attempt at combinatorial parsing. If, instead, evidence for morphological decomposition could be found, it would lend stronger support to a combinatorial mechanism.

Indeed, a number of studies have explored morphological decomposition using behavioural psycholinguistic techniques. In a lexical decision task using a repetition priming paradigm, it was shown that the presentation of either the first or second constituent as a lexical prime speeded up lexical decisions for both opaque and transparent compounds indicating constituent access for each type (Libben, Gibson, Yoon, & Sandra, 2003). This finding fits well with an earlier study showing that both opaque and transparent compounds primed lexical decision times to both first and second constituents (Zwitserlood, 1994). Decomposition has also been investigated by manipulating lexical frequencies, capitalising on the well-established lexical frequency effect observed in various paradigms in which recognition times are faster for higher-frequency lexical items. In these studies, frequency effects of both the first and second constituents were found on lexical decision times to English compounds (Andrews, 1986), although in one study the effect was greater for the second constituent (Juhasz, Starr, Inhoff, & Placke, 2003), and in a study on Spanish and Basque only the frequency of the second constituent had an effect on lexical decision times (Duñabeitia, 2007). Also in support of individual constituent access is evidence that response times to reject pseudo-compounds in a lexical decision task were longer when the individual constituents were real words (Andrews, 1986; Taft & Forster, 1976).

Other studies exploring morphological decomposition have measured eye movements during reading, which, as a continuous behavioural measure, have the potential to reveal more about the time course of lexical processing than response times alone. First-constituent frequency has been repeatedly shown to have a rapid but lasting effect on eye movements as judged by an effect on first fixation and gaze durations, whereas second constituent frequency is usually important later affecting gaze duration (Andrews, Miller, & Rayner, 2004; Hyönä & Pollatsek, 1998; Juhasz et al., 2003; Pollatsek, Hyönä, & Bertram, 2000). Although suggestive of access to both constituents, these studies notably used only transparent compounds, making it impossible to judge whether constituent access may also take place for non-transparent cases. In those few studies that have explicitly compared processing of transparent and opaque compounds (frequencies of constituents and whole-word forms were matched between the two types), no differences were obtained on any eye movement measure for either English (Frisson, Niswander-Klement, & Pollatsek, 2008) or Finnish (Pollatsek & Hyönä, 2005) stimuli, from which it was argued that morphological decomposition occurs for both types. However, in contrast to the above eye tracking findings other research has failed to show an impact of constituent frequency on lexical decision times in Dutch, but did observe whole-word frequency effects (Van Jaarsveld & Rattink, 1988), argued to reflect the absence of morphosyntactic decomposition but access to a full representation instead. Similarly, a study conducted in Chinese, this time in the auditory domain, showed that the frequency of the whole word rather than constituents could be the dominant factor affecting lexical decision times to semantically transparent compound words, which also supports access to a full-word representation (Zhou & Marslen-Wilson, 1994).

Several studies provide neurophysiological evidence for morphological decomposition. In an EEG study exploring the processing of both opaque and transparent spoken compounds, syntactic gender disagreements between the determiner and both constituents elicited a left anterior negativity (LAN) for both compound types, supporting morphological decomposition in each case (Koester et al., 2004). In a more recent study, transparent compound words were presented visually in the context of a lexical decision task whilst MEG was recorded (Fiorentino & Poeppel, 2007). Reaction times to correctly identified words were faster for compound than monomorphemic words, and even faster for those that had a high lexical frequency. The results were interpreted as reflecting access to constituents, which facilitated whole-compound processing. Analysis of the MEG data focused on the latency of the M350, a component which had previously been shown to be sensitive to lexical variables (Embick, Hackl, Schaeffer, Kelepir, & Marantz, 2001; Pylkkanen, Stringfellow, & Marantz, 2002). In line with the behavioural results, the M350 peak occurred earlier for compounds relative to monomorphemic controls, which was argued to reflect the facilitatory effect of accessing individual morpheme constituents on access to the full compound word representation.

Two recent studies attempted to measure the combinatorial process itself, using the N400 brain response as an index of lexico-semantic integration of compound constituents. Transparent compounds elicited a larger N400 than opaque compounds suggesting processing via a combinatorial mechanism (Koester et al., 2007). Focusing only on transparent compounds, a larger N400 was found for the plausible second constituents, reflecting greater integration difficulty that started even before the end of the final constituent (Koester et al., 2009). Note that although it has been argued that the M350 and N400 reflect lexico-semantic access and integration at ∼350–400 ms, there is ample evidence that these processes commence much earlier, within 200 ms, both for written (Hauk, Davis, Ford, Pulvermüller, & Marslen-Wilson, 2006) and spoken (MacGregor, Pulvermüller, van Casteren, & Shtyrov, 2012; Pulvermüller et al., 2001; Shtyrov, 2010; Shtyrov & Pulvermüller, 2002) presentations. In contrast to later, secondary stages reflected in the M350 and N400 that may be under attentional control and possibly linked to conscious (re-)analysis of the input, this early stage appears to be largely automatised and attention-independent (Pulvermüller et al., 2001; Shtyrov, 2010). It seems possible, therefore, that the neurolinguistic processes tracked by the M350 and N400 do not reveal the stages of compound access in full, missing its earliest steps in particular. These steps may be revealed by experimental designs focussed on the earliest automatic neurophysiological indices of language processing.

To summarise, the picture that emerges from the research findings on compound word processing is incomplete and not always consistent. There is some evidence that individual constituents are morphologically decomposed (Fiorentino & Poeppel, 2007; Hyönä & Pollatsek, 1998; Juhasz et al., 2003; Koester et al., 2004; Libben et al., 2003; Zwitserlood, 1994) and that transparent constituents are semantically accessed (Pratarelli, 1995; Sandra, 1990; Zwitserlood, 1994). From this it has been inferred that the processing of at least transparent compounds operates combinatorially, but only two studies (Koester et al., 2007, 2009) have attempted to measure the combinatorial process itself. There is also fragmented evidence that compounds may have a unitary lexical representation (Van Jaarsveld & Rattink, 1988; Zhou & Marslen-Wilson, 1994), but this question has not been fully investigated. In addition, most studies have presented compounds visually, thus proposed accounts may not necessarily extend to auditory presentation, the native modality of language. In the auditory domain, information about the words extends temporally over time, with the first constituent arriving before the following ones, which may promote combinatorial processing to a greater extent than when words are presented visually all at once. The current study investigated the neural processing of compounds with systematically varied psycholinguistic features, which were presented auditorily to participants whose brain activity was assessed using EEG. In a clear extension to previous studies, we used a design that enabled us to investigate the earliest and possibly automatic stages of compound word processing, which may have been missed in previous neurophysiological studies due to stimulus variability or the specific tasks used. The main questions we asked are whether processing of compound words takes place via a combinatorial or whole-form route and whether processing is influenced by the transparency and lexical frequency of the words.

A unique opportunity to address these questions neurophysiologically is offered by a brain response called the auditory Mismatch Negativity (MMN), a component of event-related brain potentials which has been shown to demonstrate markedly different behaviour in response to unified lexical entries as opposed to syntactic sequences (for a review, see Pulvermüller & Shtyrov, 2006; Shtyrov & Pulvermüller, 2007b). The MMN is elicited in response to rare (deviant) acoustic stimuli randomly presented in a context of frequent (standard) stimuli (Naatanen & Alho, 1995). Importantly, the MMN can be elicited even in the absence of active attention to auditory stimuli and can therefore be used for investigating the nature of lexical representations and processing in the brain without the need to employ tasks such as lexical or semantic decision (for a review, see Pulvermüller & Shtyrov, 2006; Shtyrov & Pulvermüller, 2007b). A large body of work has demonstrated that the MMN (difference between the standard and deviant stimuli) is enhanced for meaningful words compared to meaningless pseudo-words (Endrass, Mohr, & Pulvermüller, 2004; Pettigrew et al., 2004; Pulvermüller, Shtyrov, Kujala, & Naatanen, 2004; Pulvermüller et al., 2001; Shtyrov, Hauk, & Pulvermüller, 2004; Shtyrov, Pihko, & Pulvermüller, 2005; Shtyrov & Pulvermüller, 2002; Sittiprapaporn, Chindaduangratn, Tervaniemi, & Khotchabhakdi, 2003) and more recently, for more frequent compared to less frequent words (Alexandrov, Boricheva, Pulvermüller, & Shtyrov, 2011; Shtyrov, Kimppa, Pulvermüller, & Kujala, 2011). This “lexical MMN” enhancement is thought to index automatic neural activation of existing unitary memory representations of known words that are stronger for more frequently used items than infrequent ones, and not present for meaningless pseudo-words. It has been argued that the robustness of these representations can explain their automatic activation even in a passive listening setting, where attention is not focused on the verbal input (Garagnani, Shtyrov, & Pulvermüller, 2009; Garagnani, Wennekers, & Pulvermüller, 2008; Shtyrov, Kujala, & Pulvermüller, 2010).

In addition to its sensitivity to the lexical status of single words, the MMN is also sensitive to phrasal or combinatorial processing. A syntactically coherent sequence of words elicits a relatively small MMN relative to a syntactically illegal string of words (Hasting, Kotz, & Friederici, 2007; Menning et al., 2005; Pulvermüller & Assadollahi, 2007; Pulvermüller & Shtyrov, 2003; Pulvermüller, Shtyrov, Hasting, & Carlyon, 2008; Shtyrov, Pulvermüller, Naatanen, & Ilmoniemi, 2003). This “syntactic MMN” depends on phrasal congruity not sequential probability (Pulvermüller & Assadollahi, 2007). A similar MMN reduction is also found when words in a phrase semantically match compared to mismatch in their meaning (Shtyrov & Pulvermüller, 2007a). Such a combinatorial MMN pattern is found in passive listening designs without a stimulus-related task and is thought to reflect automatic priming between related morphemes in the syntactically and semantically congruent cases (Pulvermüller & Shtyrov, 2003).

Thus, the opposite patterns of the monomorphemic lexical MMN (meaningful/frequent word > meaningless/less frequent word) and combinatorial MMN (congruous < incongruous combination) provide a double dissociation that can be used to investigate the representation and processing of morphosyntactically complex cases such as compound words, whose linguistic status as single lexical entities or as syntactic combinations of two lexical items is unknown (cf. Cappelle, Shtyrov, & Pulvermuller, 2010). This, as we suggest below, can be done by modulating the frequency and internal congruity of compound word stimuli.

### 1.1 The present study

We investigated the representation and processing of spoken compound words using the MMN passive-listening oddball paradigm. We capitalised on the known different patterns of MMN amplitudes, asking whether hearing the second constituent of an opaque or transparent compound triggers access to a unitary representation of the whole compound or combinatorial processing.

We presented compound words (e.g. *housework*) auditorily as an infrequent deviant stimulus in the context of monomorphemic standard stimuli, which were varied over the course of the experiment. To rule out acoustic confounds and possible diverging effects of constituent frequency, we used a single second constituent (*work*) that was identical across all stimuli, whilst tightly controlling acoustic and psycholinguistic features of the first constituents in a group of 10 compound words. The two constituents formed a meaningful transparent (e.g. *homework*) or opaque (e.g. *framework*) compound word, or a meaningless pseudo-compound (*houndwork*, *grousework*). Whole-form frequency of the compounds was systematically varied over both opaque and transparent groups of stimuli. If a compound is represented as a single lexical unit as predicted by full-listing models (Bybee, 1995), we would expect a larger MMN response relative to the pseudo-compounds and a larger MMN response for more frequently occurring compounds. On the other hand, if a compound is processed by accessing the individual constituents and processing them in a combinatorial fashion, as proposed by full-parsing models (Libben et al., 1999; Taft, 2004; Taft & Forster, 1976), we would expect to see a reduced MMN response relative to the pseudo-compounds. Finally, it is possible that whole-form and combinatorial processing are recruited to different extents in the perception of opaque compared to transparent, and frequent compared to infrequent words, as would be predicted by dual-route models (Baayen et al., 1997; Isel et al., 2003; Koester et al., 2004, 2007, 2009; Sandra, 1990; Zwitserlood, 1994). In this case we would expect a complex pattern of response amplitudes depending on the particular combinations of these psycholinguistic features.

## 2 Methods

### 2.1 Participants

Twenty right-handed (according to the Edinburgh inventory, Oldfield, 1971) native British English speakers (7 male, mean age 24 years; range 19–36 years) with normal hearing and no record of neurological diseases took part in the study for financial compensation. Ethical approval was issued by the Cambridge Psychology Research Ethics Committee (University of Cambridge) and informed written consent was obtained from all volunteers.

### 2.2 Stimuli

As stimuli (see Table 1), we chose a set of acoustically similar transparent (*n* = 5) and opaque (*n* = 5) compound words, all ending in the same second constituent *work*. Based on standard dictionary definitions, the meanings of the transparent compounds could be derived by combining the meaning of the two constituents (e.g., *home* + *work* means work done at home), whereas this was not the case for opaque compounds (e.g., *ground* + *work* means preliminary or preparatory work, not work done on the ground). The opaque and transparent stimulus groups were matched on their frequency of occurrence per million words using the CELEX database (Baayen, Piepenbrock, & Gulikers, 1995). Within each group, frequency was graded systematically from high to low; subgroups of high- and low-frequency compounds – matched between the groups – each contained two compounds. In addition, there were two pseudo-compounds also ending in *work*.

**Fig. 1:**
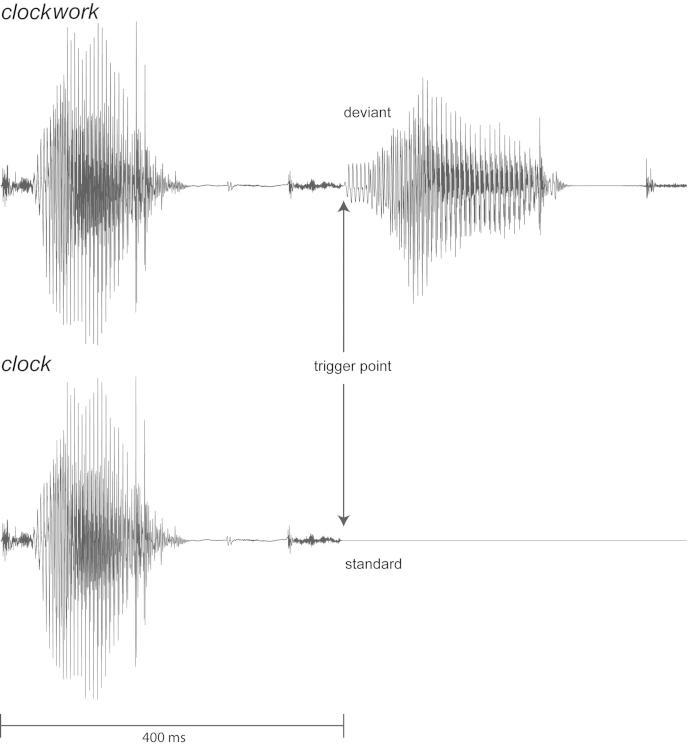
Acoustic stimuli and ERP time locking. Example waveforms of acoustic stimuli for the deviant and standard conditions for one of the compound words (clockwork). Stimuli are identical up to the divergence point, when *work* starts and it becomes clear the stimulus is a compound. ERPs were formed time-locked to this point. Acoustically identical tokens of *work* were used across all compound words.

To create the stimuli, multiple repetitions of all constituents were uttered by a female native speaker of British English. We also recorded examples of complete compound words for comparison with our final experimental compounds to ensure a natural prosodic pattern associated with compound words. We selected a set of first-constituent recordings with similar fundamental frequencies (F0) and overall durations, and a recording of the second constituent *work* that, when combined with the first constituents, fulfilled the criterion of a natural prosody whereby the first constituent had a stronger accent (mean average root-mean-square (RMS) power: −20 dB vs. −26 dB) and higher pitch (mean F0: 233 vs. 172 Hz) than the second constituent. The stimuli were edited to have the same mean sound energy in the first constituent, by matching the average RMS power of the acoustic signal, and were made identical in their duration (400 ms per constituent) by removing extraneous short sections on their flanks. Finally, these were cross-spliced to form 12 compounds (Table 1) of 800 ms duration. This strictly controlled stimulus set allowed us to time-lock neural responses to the onset of the second constituent (*work*) because this is the first point in time where the standards (e.g., *school*) differ from the deviants (e.g., *schoolwork*) and thus (1) the earliest point in time at which the compound could be recognised (although it may still be later, but not earlier than this divergence point), and (2) the earliest point in time when the standard and deviant stimuli diverge and thus the MMN response *per se* can be triggered. Critically, the second constituent was the same acoustic token across all conditions (Fig. 1), implying that the physical/acoustic standard-deviant contrast was always identical. Thus, the purely acoustic component of the MMN is expected to be identical, and ERP differences between MMNs in different conditions can be due only to the impact of the linguistic context on neural word processing. Furthermore, any contribution of the first constituent *per se* to the responses would be removed by the calculation of the MMN (deviant minus standard operation) – the dependent variable in the experiment. Thus, we could attribute any differences in MMN amplitude to our experimental linguistic manipulations of semantic transparency and frequency across conditions.

### 2.3 Procedure

Each compound was presented as an infrequent (deviant) stimulus (probability of 18%) amongst a frequently occurring (standard) stimuli which were the respective first constituents presented in isolation in each of the different 12 blocks (e.g., standard *house*, deviant *housework*). Auditory stimuli were presented, with a stimulus onset asynchrony of 1400 ms (jittered ±80 ms in 10 ms steps), via headphones in a passive-listening paradigm at 50 dB above individual hearing threshold determined before the start of the experiment. Participants were told to focus their attention on a film, which they were free to choose, and to ignore the auditory stimuli. Standards and deviants were presented in a random sequence with the constraints that a deviant was always followed by at least one standard (which was not included in the analysis) and never by another deviant.

### 2.4 EEG recording and data processing

EEG was recorded (Brain Products Recorder) from 128 Ag/AgCl active electrodes (actiCAP, Brain Products GmbH) embedded in a cap based on the 5% positioning system (Oostenveld & Praamstra, 2001). Data were recorded using the FCz as reference and AFz as ground. Monopolar EOG electrodes, including FP1, were used to monitor for vertical eye movements, and F10 and F9 served to monitor for horizontal eye movements. Electrode impedances were kept below 5 kΩ. The analogue EEG recordings were amplified (band pass filter 0.1–100 Hz), and continuously digitised (16-bit) at a sampling frequency of 500 Hz.

Data were processed offline using Brain Vision Analyser (1.0). Bipolar electro-oculogram channels were reconstructed for vertical (VEOG) and horizontal (HEOG) eye movements from monopolar EOG recordings (e.g. HEOG = F10 minus F9) and data were filtered (0.1–30 Hz, 12 dB/Oct, Butterworth zero phase filter). Continuous data were visually inspected, and bad channels (mean of 1 channel per participant) interpolated using the average of the surrounding channels. Data were corrected for eye blink artifacts (Gratton, Coles, & Donchin, 1983) and re-referenced to the average of all 125 scalp electrodes (i.e., excluding EOGs). Data were epoched between −50 and 600 ms relative to the onset of the markers (onset of *work* for the deviants or corresponding silent period for the standards) and baseline corrected over the 50 ms pre-stimulus period. The epoched data were rejected if the difference between the minimum and maximum amplitudes within the 650 ms epoch exceeded 100 μV, if absolute amplitude exceeded ±60 μV, if amplitude exceeded 50 μV between consecutive sampling points, or if the difference between the maximum and minimum amplitudes was less than 0.5 μV in a 100 ms period. The screening process resulted in the loss of 13% of the trials for both standards and deviants. Two participants who produced a larger number of artifacts were excluded, and thus data are reported for the remaining 18 participants. For each compound and each participant, average ERPs were formed for the standards and deviants separately (comprising a mean of 153 and 43 trials per average respectively) and MMN responses computed by subtracting ERPs to standards from those to the deviants. Grand average MMN responses were calculated across all participants and compared between experimental conditions.

### 2.5 EEG data analysis

Overall activation strength of the ERPs was first quantified as the global root mean square (RMS) of the MMN responses across the 125 scalp electrodes: the grand average MMN response was calculated across all compounds and participants for each electrode, then for each time point the square root was calculated for the mean of the squares across electrodes. The amplitude of the ERP brain responses to the different compound words were compared between experimental conditions with repeated measures Analyses of Variance (ANOVAs) using the Greenhouse-Geisser correction for inequality of variance where appropriate (data are reported with corrected *p* values). For statistical analysis, mean amplitudes were calculated over time windows selected to capture the prominent effects based on peaks of activation in the global RMS. To explore the data fully, ANOVAs were performed on data from electrodes across the scalp, which were grouped into 6 clusters (of 6 electrodes) arranged according to Location (frontal, central, parietal) and Hemisphere (left, right). An initial analysis included the factors of Frequency (5 levels of frequency) and Compound Type (opaque vs. transparent). Since overall frequency was matched across the two compound types but not on a word by word basis, for subsequent analyses we split the words into a high frequency group and a low frequency group for transparent and opaque compounds separately to investigate possible interactions between the two factors of interest and to compare the effects for meaningful compounds against those for pseudo-compounds. Each stimulus group therefore comprised the mean of two compounds.

In addition to the signal-space ERP analysis, an L2 minimum-norm current estimation was also attempted; as the critical predictions and the focus of the current study is on the relative size of the MMN amplitude in signal space, this preliminary grand-average based source analysis was included only as a point of secondary interest and is thus presented in the Supplementary data only (see Supplementary Methods, Results and Fig. S1).

## 3 Results

**Fig. 2:**
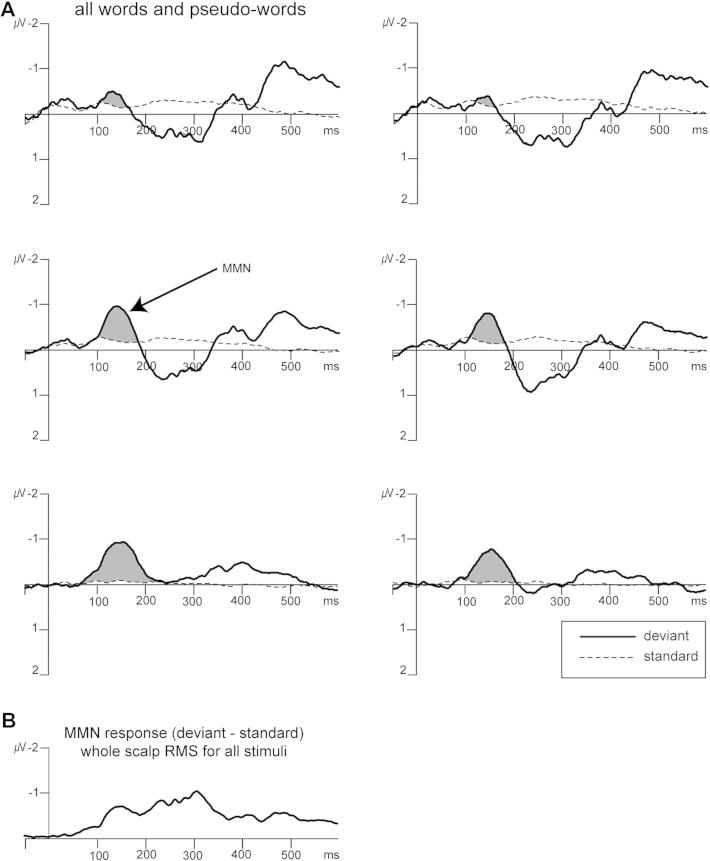
MMN responses for all compound word stimuli. The upper panel A shows the grand average ERPs (*n* = 18) elicited in response to deviant (solid line) and standard (dotted line) stimuli calculated across all 10 compounds at the six electrode clusters. ERPs display a typical MMN response, greater negativity for deviants compared to standards, which is highlighted in grey. The lower panel B shows the global root mean square (rms) of the MMN responses (deviant minus standard). The RMS was calculated over all 125 scalp electrodes, 10 compound words, and participants (*n* = 18). Peaks of activation were used to define time windows for analyses that compared the effects between conditions.

**Fig. 3:**
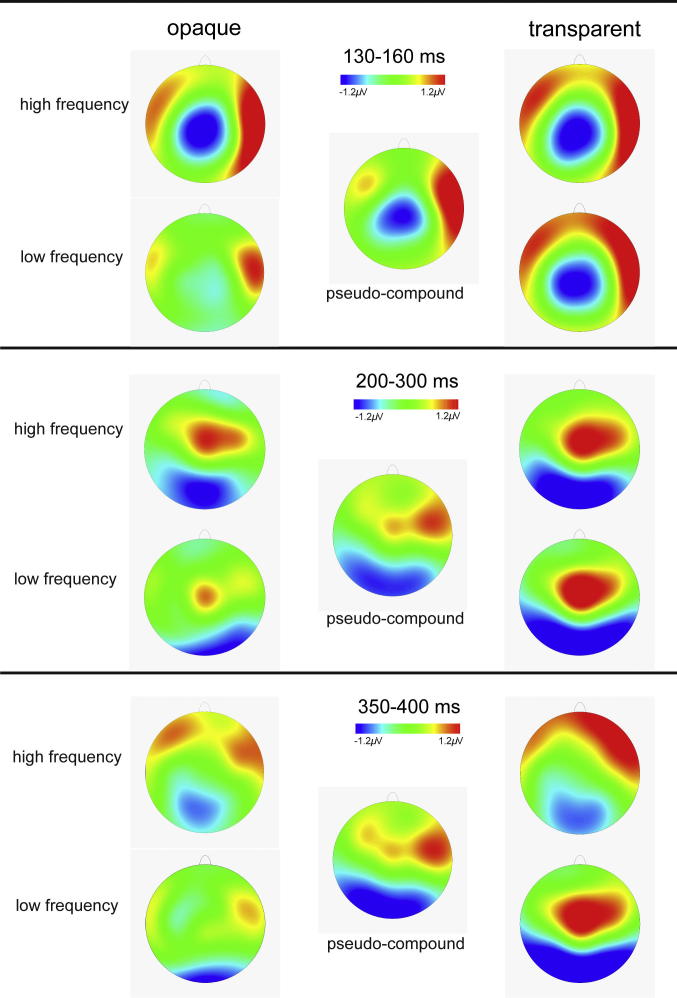
MMN response distributions. Topographic maps show the distribution of the MMN responses (deviant minus standard) over the three time windows that were analysed, separately for compounds of different types and frequencies, and for the pseudo-compounds.

All compound words elicited an MMN response – increased negativity for deviants relative to standards (Fig. 2) – particularly over central locations (Fig. 3). As expected, the global RMS (Fig. 2) indicated a prominent peak of activation within the typical MMN time range, and thus analyses comparing the MMN amplitude between experimental conditions were performed on the mean MMN amplitude calculated over a 30-ms time window (130–160 ms) around its peak. In addition, there was a broad increase in positivity around 200–300 ms, particularly over fronto-central regions, and around 350–400 ms over posterior scalp regions (see Figs. 2 and 3). Although we did not have specific hypotheses regarding the ERPs at these time points, these effects correspond closely in timing and topography to the P3a and the N400 and additional analyses were performed to compare these later effects between conditions using the mean amplitude calculated over 200–300 ms (P3a) and 350–400 ms (N400).

### 3.1 MMN effects

The first analysis on all 10 compounds with the factors of Type (transparent vs. opaque), Frequency (5 levels), Location (frontal vs. central vs. parietal) and Hemisphere (left vs. right) revealed a main effect of Type [*F*(1, 17) = 7.716, *p* = .013], a main effect of Frequency [*F*(4, 68) = 3.148, *p* = .037], and an interaction between Type and Frequency [*F*(4, 68) = 3.200, *p* = .027].

This general pattern was confirmed by a subsequent analysis with the factors of Type (opaque vs. transparent), Frequency (high vs. low), Location (frontal vs. central vs. parietal) and Hemisphere (left vs. right). A four-way interaction between Type, Frequency, Location and Hemisphere [*F*(2, 34) = 4.542, *p* = .032] indicated that the effect of Frequency differed as a function of the type of compound (transparent or opaque) and thus to explore this interaction further, separate ANOVAs were performed for opaque and transparent compounds, with factors of Frequency (high vs. low), Location (frontal vs. central vs. parietal) and Hemisphere (left vs. right). The mean MMN amplitudes for the different conditions are shown in Table 2.

**Fig. 4:**
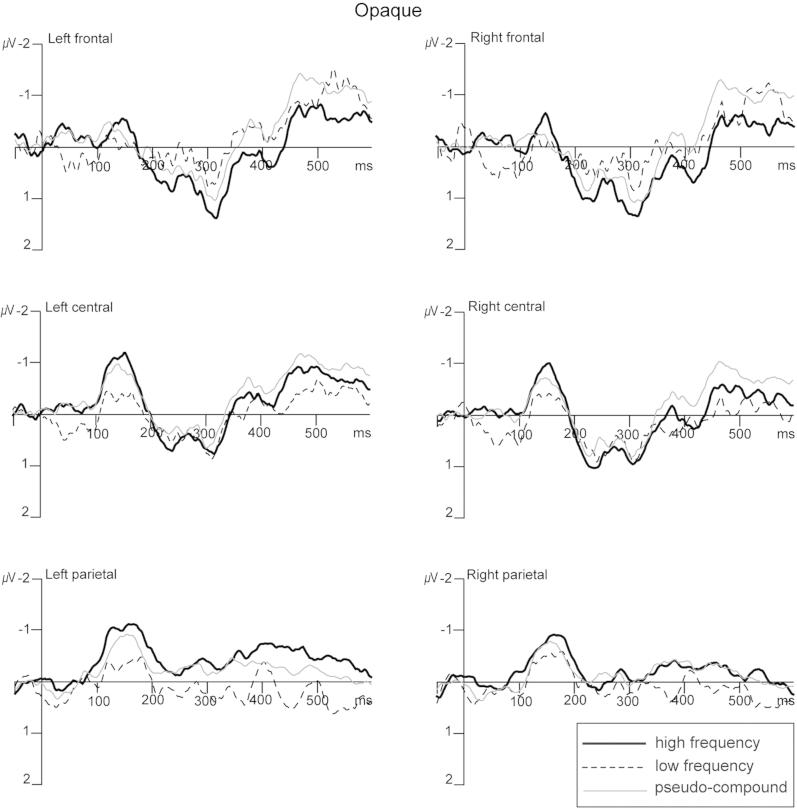
MMN responses showing frequency and lexicality effects for opaque compounds. Grand average (*n* = 18) MMN responses (deviant minus standard) elicited relative to the second constituents (i.e. *work*) of meaningful high frequency (black solid line) and low frequency (dotted line) compounds, and pseudo-compounds (grey line). Data are shown for the six electrode clusters used in the analyses.

For opaque compounds (Figs. 4 and 2 right panel) there was a main effect of Frequency [*F*(1, 17) = 5.287, *p* = .033] but this was qualified by an interaction between Frequency, Location and Hemisphere [*F*(2, 34) = 5.404, *p* = .021]. Follow-up ANOVAs for each location separately indicated that high frequency compounds were associated with a significantly larger MMN response than low frequency compounds at central locations [*F*(1, 17) = 6.066, *p* = .025]. At parietal locations, an interaction between Frequency and Hemisphere [*F*(1, 17) = 5.917, *p* = .026] reflected a greater MMN response for high frequency compounds over the left location [*F*(1, 17) = 10.265, *p* = .005]. There was no frequency effect at the frontal location. Focusing then on the central and parietal regions where the frequency effect was maximal, we ran an ANOVA with factors of Condition (high frequency vs. low frequency vs. pseudo-compound), Location (central vs. parietal) and Hemisphere (left vs. right), which revealed a main effect of Condition [*F*(2, 34) = 4.580, *p* = .026]. This reflected a larger MMN response for high compared to low frequency compounds [*F*(1, 17) = 6.679, *p* = .019], particularly over the left hemisphere [*F*(1, 17) = 4.750, *p* = .044], and a trend towards a larger MMN for pseudo-compounds than low frequency compounds [*F*(1, 17) = 3.180, *p* = .092].

**Fig. 5:**
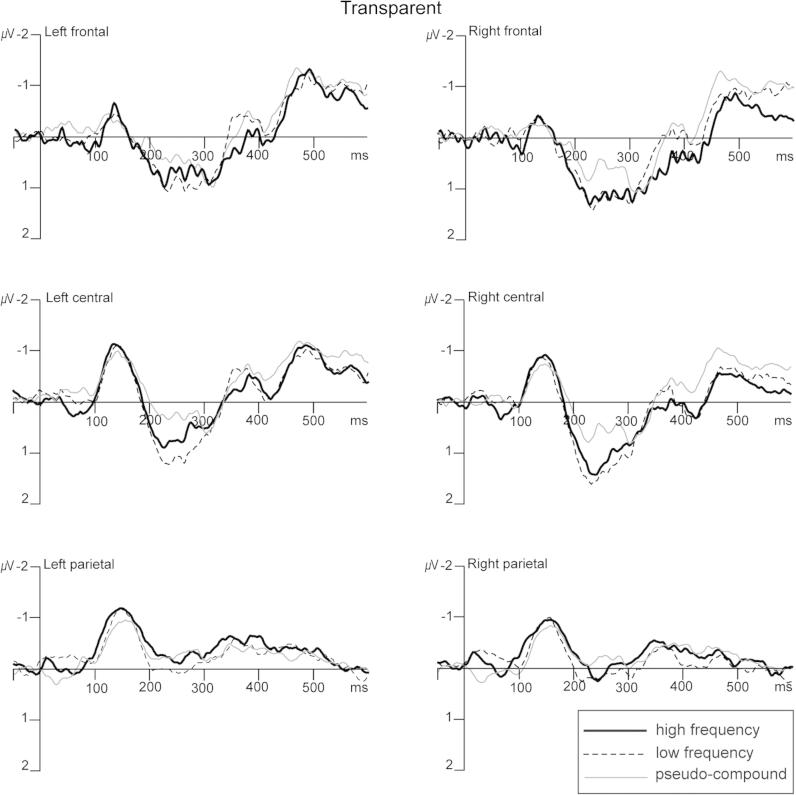
MMN responses showing lack of frequency and lexicality effects for transparent compounds. Grand average (*n* = 18) MMN responses (deviant minus standard) elicited relative to the second constituents (i.e. *work*) of meaningful high frequency (solid line) and low frequency (dotted line) compounds, and pseudo-compounds (grey line). Data are shown for the six electrode clusters used in the analyses.

By contrast, analysis of the transparent compounds (Figs. 5 and 2 right panel), indicated no effect of frequency on MMN response amplitude [*F* < 1]. An ANOVA to compare the high and low frequency transparent compounds with the pseudo-compounds with factors of Condition (high frequency vs. low frequency vs. pseudo-compounds), Location (frontal vs. central vs. parietal) and Hemisphere (left vs. right) also revealed no differences in MMN response amplitude between the conditions, nor did an ANOVA focusing only on central and parietal locations [*F*s < 1].

### 3.2 Post-MMN effects

Across all conditions, following the observed MMN response, there was an increased positivity for the deviant compared to the standard stimuli at around 250 ms, which appeared over frontal and central scalp regions (Figs. 2 and 3). This was followed by an increase in negativity at around 350–400 ms, which appeared over posterior scalp regions but was also observed to an extent at central regions (Figs. 2 and 3).

#### 3.2.1 P3a: 200–300 ms

The first analysis on all 10 compounds with the factors of Type (transparent vs. opaque), Frequency (5 levels), Location (frontal vs. central vs. parietal) and Hemisphere (left vs. right) revealed an interaction between Type and Frequency [*F*(4, 68) = 7.675, *p* < .001]. A subsequent analysis with the factors of Type (opaque vs. transparent), Frequency (high vs. low), Location (frontal vs. central vs. parietal) and Hemisphere (left vs. right) revealed a main effect of Type [*F*(1, 17) = 7.036, *p* = .017], reflecting larger positivity for transparent (.684 μV) than for opaque (.469 μV) compounds.

#### 3.2.2 N400: 350–400 ms

The first analysis on all 10 compounds with the factors of Type (transparent vs. opaque), Frequency (5 levels), Location (frontal vs. central vs. parietal) and Hemisphere (left vs. right) revealed a marginal effect of Type [*F*(1, 17) = 3.517, *p* = .078] reflecting greater overall negativity for transparent (−.173 μV) compared to opaque (−0.14 μV) compounds. There was also an interaction between Frequency and Location [*F*(8, 136) = 3.486, *p* = .009]. Similarly, a subsequent analysis with the factors of Type (opaque vs. transparent), Frequency (high vs. low), Location (frontal vs. central vs. parietal) and Hemisphere (left vs. right) also revealed an interaction between Frequency and Location [*F*(2, 34) = 7.396, *p* = .008]. Follow-up analyses at each location separately confirmed that the interaction reflected a larger negativity for low frequency (−.089 μV) compared to high frequency (.299 μV) compounds at frontal locations [*F*(1, 17) = 4.960, *p* = .040]. Effects at central and parietal locations were not significant. Opaque and transparent compounds were analysed separately to assess whether effects for the real word compounds differed to pseudo-compounds. For transparent compounds, an analysis with factors of Condition (high frequency vs. low frequency vs. pseudo-compounds), Location (frontal vs. central vs. parietal) and Hemisphere (left vs. right) revealed a marginal interaction between Condition and Location [*F*(4, 68) = 2.843, *p* = .076]. Follow up analyses at each location separately showed that at the frontal location there was a main effect of Condition [*F*(2, 34) = 3.978, *p* = .030], reflecting greater negativity for pseudo- (−.169 μV) compared to high frequency (.309 μV) compounds [*F*(1, 17) = 6.644, *p* = .020]. There was no statistical difference between pseudo-compounds and low frequency compounds and effects at central and parietal locations were not significant.

## 4 Discussion

The aim of the present study was to investigate whether compound words are represented lexically as a unified form or processed combinatorially from the two constituents. We recorded, in a passive-listening oddball paradigm, electrical brain responses to stimuli which, in their presentation context, formed a meaningful compound word that was opaque or transparent, or a meaningless pseudo-compound. Orthogonally to transparency, the compound lexical-frequency was systematically modulated. In all conditions the words elicited an MMN response, which peaked at ∼150 ms over midline sites, and varied in amplitude depending on compound type (transparent or opaque) and on frequency. Differences between conditions were also observed in two later time windows, corresponding to the P3a and the N400. The key findings are discussed below in more detail.

### 4.1 Lexical access and combinatorial processing depends on compound frequency and transparency: MMN evidence

For opaque compounds only, there was a larger MMN for high frequency than low frequency compounds. This finding extends previous findings of word frequency effects on the MMN amplitude for monomorphemic words (Alexandrov et al., 2011; Pulvermüller et al., 2001; Shtyrov et al., 2011) to a specific category of polymorphemic items. Drawing on these previous studies, we interpret the frequency-based enhancement of the MMN as reflecting stronger unified lexical representations for more frequently used opaque compounds and only a very weak representation for low frequency compounds. No such frequency effects are known to exist for syntactic MMNs (Pulvermüller & Assadollahi, 2007) and thus the results suggest a dominant role of lexical storage whereby meaning is more likely to be accessed from a unitary representation than computed via a combinatorial mechanism.

The lexical MMN has previously been explained neurobiologically as reflecting the activation of a strongly integrated network of neurons formed through associative learning mechanisms. Over time, as words are used more frequently and become more familiar, the strength of mutual connections between simultaneously active network nodes increases (Pulvermüller et al., 2001). Upon hearing a highly frequent known word, there is an ‘ignition’ of the word network which is realised as an increased neural response. By contrast, the activation level invoked by a less frequent word with a weaker representation will be lower. Future work may consider how such representations develop over time as the meanings of novel compounds are acquired in the learning process.

In addition to the signal-space ERP analysis, an L2 minimum-norm current estimation was also attempted (see Supplementary Methods). These preliminary localisation results (see Supplementary Results and Fig. S1) suggested that the main generators of the MMN frequency effect for opaque compounds were bilaterally located in the inferior frontal lobes and superior temporal lobes, although maximal on the left. The source of the difference between pseudo-compounds and low frequency opaque compounds was also bilateral. Although frequency effects have been observed previously for monomorphemic words in the left inferior frontal and temporal lobes in EEG (Shtyrov et al., 2011) as well as fMRI (Carreiras, Mechelli, & Price, 2006), in the present study the frequency effect was also present on the right suggesting a more distributed (and bilateral) nature of complex word processing. We note that the grand average source distributions reported here have to be treated with caution, in the absence of statistics. The focus of the current paper is on the relative size of the MMN amplitude; future work using individual neuroanatomical MR images, and high-density MEG or combined EEG-MEG could address the question of brain generators of the current effects.

The lack of frequency effects for transparent compounds is clear evidence against a purely lexical account of transparent compound processing (which would predict a lexical MMN: high frequency > low frequency compounds > pseudo-compounds). At the same time, the lack of difference between transparent compounds (both high and low frequency) and pseudo-compounds speaks against a purely combinatorial processing route (which would predict a syntactic MMN advantage for incongruous combinations of the pseudo-compounds: transparent < pseudo-compounds). Taken together, the similar MMN responses for high frequency, low frequency, and pseudo-compounds suggest that the meaning of transparent compounds is computed online from the individual constituents via a combinatorial mechanism but that there is also access to a unitary lexical representation. Such a dual-route mechanism has been suggested previously by a number of studies (Baayen et al., 1997; Isel et al., 2003; Koester et al., 2004, 2007, 2009; Sandra, 1990; Zwitserlood, 1994).

### 4.2 Attentional orientation may be greater during combinatorial processing: evidence from the P3a

Although the experimental predictions and design focused on the MMN response, we also observed later shifts in ERP amplitude. Following the MMN, there was an increase in positivity around 200–300 ms which appeared over frontal and central scalp regions. The timing and topography of the effect are compatible with its classification as a P3a (Squires, Squires, & Hillyard, 1975), also referred to as the novelty P3 (Simons, Graham, Miles, & Chen, 2001; Spencer, Dien, & Donchin, 2001), which is often observed following an MMN and thought to reflect automatic attentional re-orientation or novelty detection in the absence of direct attention. This P3a was enhanced for transparent compared to opaque compounds, and localised predominantly to left frontal and inferior frontal cortex (see Fig. S1). Based on previous studies of the P3a, we suggest that automatic attentional orientation is greater when processing of compounds relies more on combinatorial processing as in the case of transparent compounds. Interestingly, previous MMN studies of monomorphemic words which indexed lexical access typically did not show a similarly clear P3-like effect in passive listening conditions. Thus its presence here might also be an indicator that combinatorial processing of compound words requires more in-depth processing, which recruits stronger attentional resources, than monomorphemic words. P3a-like effects are, however, elicited by spoken stimuli when they are attended to (Shtyrov et al., 2010, 2012). Furthermore, a P3a-like effect appeared in a passive-listening syntactic MMN study (Pulvermüller et al., 2008), implying an increase in attentional demand for combinatorial processing. In the context of the current study, this would also speak in favour of a predominantly combinatorial route for transparent compounds. No P3a effects were found for the opaque words implying a distinct processing route (as well as different attentional demands) for these stimuli. The role of attention and its potentially differential involvement in processing monomorphemic and bimorphemic words as well as word strings could be tested in future experiments.

### 4.3 Semantic integration processes during combinatorial processing of transparent compounds: Evidence from the N400

Following the P3a, there was an increase in negativity around 400 ms, which was larger over frontal locations for transparent compared to opaque compounds, for low- compared to high-frequency compounds, and for pseudo-compounds compared to high frequency compounds. The timing and distribution of the effect lead us to interpret it as a classic auditory N400 (Kutas & Hillyard, 1980). The N400 is typically elicited in response to meaningful stimuli and thought to reflect access or integration of conceptual information (Kutas & Federmeier, 2011), which in the case of compounds would occur during combinatorial processing, but not direct lexical access. The larger N400 for transparent compared to opaque compounds replicates previous findings using German compounds (Koester et al., 2007), suggesting combinatorial processing for transparent but not opaque compounds. Such an account is also supported by data from the processing of Chinese compounds which demonstrated an N400 reflecting semantic integration processes rather than a general increase in processing resources (Bai et al., 2008). The larger N400 for low frequency compared to high frequency compounds and for pseudo-compounds compared to transparent high frequency compounds correspond well to previously-observed frequency effects (Friedrich, Eulitz, & Lahiri, 2006; Van Petten & Kutas, 1990) and are well-explained in terms of more difficult integration during combinatorial processing of the constituents. Interestingly, no differences were observed between pseudo-compounds and the low-frequency compounds, compatible with integration difficulties in each case. The N400 between-condition differences were localised predominantly to the left temporal and inferior frontal cortices (Fig. S1), which corresponds well to previous fMRI research suggesting that these areas may have particular roles in lexico-semantic (temporal) and attentional (frontal) processes respectively (Gold et al., 2006; Kotz, Cappa, von Cramon, & Friederici, 2002; Mummery, Shallice, & Price, 1999; Rossell, Price, & Nobre, 2003).

### 4.4 Evidence for a dual-route account of compound word processing

Our data are most compatible with a dual-route account of compound word processing (Baayen et al., 1997; Isel et al., 2003; Koester et al., 2004, 2007, 2009; Sandra, 1990; Zwitserlood, 1994). In line with previous suggestions, our data indicate that lexical representations are accessed for both constituents (Andrews, 1986; Andrews et al., 2004; Duñabeitia, 2007; Hyönä & Pollatsek, 1998; Juhasz et al., 2003; Koester et al., 2004; Pollatsek et al., 2000) and combinatorial processing of meaning is attempted, with a relative preference for a particular route being dependent on the psycholinguistic properties of transparency and frequency. For opaque compounds, combinatorial processing fails to result in an interpretable meaning, leading to the suppression of individual constituent representations, and the access to a unitary representation wins out as the dominant mechanism resulting in clear lexical MMN frequency effects. For transparent compounds, computation of and access to a meaning are both viable mechanisms, with a potential shift of balance towards lexicalisation for well-known, frequently used items, and towards a combinatorial processing mechanism for the lesser known words. Importantly, the equivalent timing of the syntactic and lexical MMNs suggests that access to a unitary representation may be attempted in parallel to combinatorial processing. Specifically, our results demonstrate an effect of both combinatorial mechanisms and access to a unitary representation, within ∼150 ms after the onset of the second constituent of a compound. This is evidence against pure supra-lexical models, for example those proposed for derivational morphology (Giraudo & Grainger, 2000, 2001), which posit constituent access only after the whole word form is accessed, and only for semantically transparent morphemes. Instead, our data fit best with dual-route accounts in which combinatorial processing and direct access to a unitary representation can occur in parallel (Andrews et al., 2004; Pollatsek et al., 2000).

The results of the present study were obtained under passive listening conditions. In accordance with previous results on the lexical (Alexandrov et al., 2011; Pulvermüller et al., 2001; Shtyrov et al., 2010, 2011) and syntactic (Hasting et al., 2007; Menning et al., 2005; Pulvermüller & Shtyrov, 2003; Pulvermüller et al., 2008; Shtyrov et al., 2003) MMNs we suggest that the earliest stages of lexical access and combinatorial processing are automatic, taking place in the absence of focused attention on the spoken stimuli. Following these early processes indexed by the MMN, there is a cascade of further processes which we suggest reflect more in-depth processing, re-analysis and integration of information and, unlike the earliest steps, engage an attentional mechanism (Shtyrov, 2010). Accordingly, in the present study we observed changes to the P3a and the N400 effects,

Further investigation into the impact of the semantic relationship between the first and second constituents is warranted. Our compounds were either fully transparent or fully opaque, although in the opaque examples, there was some degree of overlap between the constituent basic sense and its meaning in the compound (e.g. *clockwork* refers to the smooth running of something, which is related to the regularity of the mechanism in a clock). However, there are other types of compounds in which the first and second constituents differ in terms of their semantic transparency with respect to the whole word, and such differences may impact on the dominant processing route. Furthermore, even for fully transparent compounds, there is a wide variety in the nature of semantic relationships between the constituents (for example, compare *football*, *baseball*, *volleyball* or *snowman*, *fireman*, *milkman*) such that the meaning is never fully predictable from its constituents (Libben et al., 2003). For this reason, compounds may differ from regular forms of other morphologically complex words such as inflections and derivations in requiring a unitary representation depending on the exact semantics in each particular case.

We chose to use the MMN paradigm because it is now well-established as a method for investigating the nature of lexical processing and linguistic representations, and in particular for revealing the earliest automatised stages of spoken language processing (Pulvermüller & Shtyrov, 2006). One confound of this approach is multiple repetition of stimuli. This is an inherent feature of the paradigm, which offers a number of methodological benefits, although it does restrict generalisability of results. As such, however, it does not present a problem for the current results. Although repetition may lead to response decrease over time due to habituation, this effect would be identical in all conditions and thus cannot explain any differences between conditions, which is where our key results were found. Similarly, any effect of experiment duration on an MMN amplitude decrease across the whole experiment would not affect between-condition differences because of the counterbalancing of the order of presentation of the specific compounds. The particular benefits of the MMN paradigm are that it allows for strict control of acoustic and phonological parameters of the stimuli, minimal stimulus variance, precise time-locking of brain responses to linguistic events, and fully matched standard-deviant acoustic contrasts between conditions. Strikingly, in spite of using such a passive-listening paradigm with repetitive stimulation, in the N400 time range our results match closely those obtained in previous studies with more active tasks, both on compounds (Koester et al., 2007) and on word lexicality (Friedrich et al., 2006) and frequency (Van Petten & Kutas, 1990), implying high degree of compatibility between this paradigm and conventionally used multi-token active tasks. Importantly, the strict control over stimulus features afforded by the MMN paradigm allowed us to shed light on additional stages occurring prior to those previously observed during the N400 time range. Still, it is important that conclusions drawn about linguistic processing on the basis of data from MMN paradigms are verified using alternative, more ecologically valid settings where listeners would seem to engage in more natural listening. Indeed, recent studies indicated that, with due control over stimulus features, both early lexical (MacGregor et al., 2012) and syntactic (Hasting et al., 2007) MMN effects can be replicated in the absence of item repetition in non-oddball designs, suggesting a strong resilience of these automatic phenomena to stimulus repetition.

### 4.5 Conclusions

The present study investigated the neural processing of spoken compounds using event-related potentials. Results are consistent with a dual-route mechanism for compound processing in which individual constituents are accessed and combinatorial processing attempted, alongside direct access to a unitary representation, which will be stronger for more frequent compounds. For opaque compounds, the direct access route is dominant (combinatorial processing will fail and access to individual constituents will be suppressed). For transparent compounds, both routes may be successful. In sum, we suggest that the case of compound words demonstrates the dynamic and flexible mechanisms supporting lexical processing in the brain.

## Figures and Tables

**Table 1:** Stimuli and lexical frequencies. Stimuli in each of the conditions and corresponding log frequencies as obtained from CELEX.

Compound	Type	Frequency	Whole form	First constituent
			Ln freq/million	Ln freq/million
Bridgework	Opaque	Low	0	7.13
Groundwork	Opaque	Low	2.71	8.08
*Mean LF* (*stdev*)			*1.35* (*1.91*)	*7.6* (*0.67*)
Patchwork	Opaque	Medium	3.61	6.38
Clockwork	Opaque	High	3.76	6.59
Framework	Opaque	High	5.78	6.71
*Mean HF* (*stdev*)			*4.77* (*1.43*)	*6.65* (*0.08*)
*Mean all* (*stdev*)			*3.17* (*2.10*)	*6.98* (*0.68*)

Deskwork	Transparent	Low	0	7.40
Schoolwork	Transparent	Low	2.71	9.13
*Mean LF* (*stdev*)			*1.35* (*1.91*)	*8.26* (*1.22*)
Teamwork	Transparent	Medium	3.40	7.50
Homework	Transparent	High	4.77	9.17
Housework	Transparent	High	4.89	9.13
*Mean HF* (*stdev*)			*4.83* (*0.08*)	*9.24* (*0.1*)
*Mean all* (*stdev*)			*3.15* (*1.99*)	*8.50* (*0.96*)

Houndwork	Pseudo	–	–	5.11
Grousework	Pseudo	–	–	4.82
*Mean* (*stdev*)				*3.34* (*1.08*)

**Table 2:** MMN amplitudes. Mean amplitudes of the MMN brain response (130–160 ms) averaged over central and parietal electrode clusters where the MMN was maximal, shown for the four main conditions of interest.

Type	Frequency	MMN amplitude (μV)
		130–160 ms
Opaque	High	−.955
Opaque	Low	−.478
Transparent	High	−.960
Transparent	Low	−.940
Pseudo		−.790

## Appendix A Supplementary material

Supplementary data 1 L2 minimum norm source estimations.

## Footnotes

1 Although compounds may have multiple constituents, 2-constutuent compounds are most common in the majority of European languages and therefore most often used in investigations.
