# Supplementary material for: Multiple routes for compound word processing in the brain: Evidence from EEG
Source: Brain Lang. 2013 Aug;126(2):217–29. doi: 10.1016/j.bandl.2013.04.002 (PMC3730057; doi:10.1016/j.bandl.2013.04.002)
Supplement: Supplementary data 1 — L2 minimum norm source estimations. [file mmc1.pdf]

Multiple routes for compound word processing in the brain: Evidence from EEG

Supplementary Methods

Following the ERP analyses, we estimated the underlying cortical sources of the between-condition effects of interest using L2 minimum-norm current estimation (L2 MNE) implemented in CURRY 6 (Compumedics Neuroscan, Hamburg, Germany). The minimum-norm method localises neural activity in the brain based on recordings at the surface of the scalp by producing a solution that models the recorded surface voltage with the smallest amount of overall activity. (Hämäläinen & Ilmoniemi, 1994; Ilmoniemi, 1993). The L2 MNE method does this by determining the combination of sources that have a minimum total power (i.e. sum of squared current amplitudes). L2 MNE solutions were calculated for the grand average ERPs rather than individual data to optimise the signal-to-noise ratio (SNR), thus statistical analyses were not performed. Reconstruction was performed using a three-layer boundary element model (BEM) with triangularised grey matter surface of a standardised brain (Montreal Neurological Institute) to account for the distortion of EEG currents by the inner skull, outer skull and skin. Solutions were restricted to the grey matter surface. In reconstruction, noise in the EEG signal was estimated using the noise covariance matrix.

Supplementary Results

In the MMN time window, results of L2 MNE estimation (Figure S1) for the opaque frequency effect indicated strongest sources in the left inferior frontal lobe, which were accompanied by left temporal sources as well as weaker activation in the right hemisphere. Similarly, source analysis of the difference between pseudo-compounds and opaque low frequency compounds was also localised to perisylvian areas bilaterally. In the P3a time

window (200-300 ms), MNE L2 source analysis indicated dominant sources of the difference between transparent and opaque compounds over left frontal and inferior frontal cortex. In the N400 time window (350 – 400 ms), the dominant source of the main frequency effect was localised to the posterior region of the left temporal lobe accompanied by right inferior frontal activation. The pattern was similar for the source of the pseudo-compound minus transparent high frequency effect, with additional source activation observable in anterior regions of the left temporal lobe. The transparent minus opaque difference was localised to the left inferior frontal cortex.

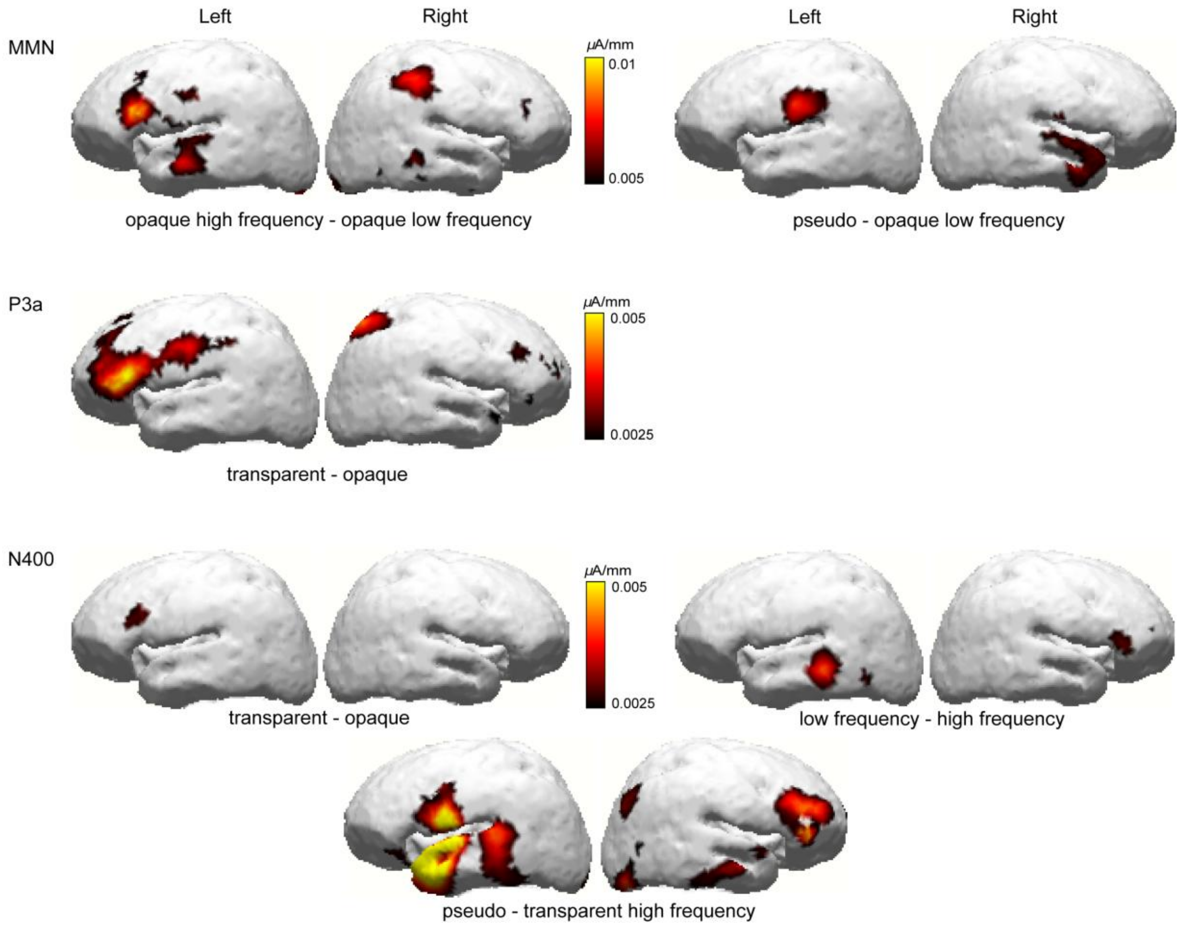

Figure S1. Cortical sources of the MMN response differences. L2 Minimum norm source estimations for the grand average (n=18) between-condition differences in the MMN responses that were identified in the channel-level analyses over the three time windows.

Supplementary References

Hämäläinen, M. S., & Ilmoniemi, R. J. (1994). Interpreting magnetic fields of the brain: minimum norm estimates. *Medical and Biological Engineering and Computing*, 32, 35-42.

Ilmoniemi, R. J. (1993). Models of source currents in the brain. *Brain Topogrography,* 5, 331-336.
